# P-1310. From Website to Parasite: An Analysis of the Unregulated Online Helminth Therapy Supply Chain

**DOI:** 10.1093/ofid/ofae631.1491

**Published:** 2025-01-29

**Authors:** Liliana J Ramirez, Tessa L Danehy, Louis M Weiss

**Affiliations:** University of Mary Washington, Newport News, Virginia; Albert Einstein College of Medicine, Bronx, New York; Albert Einstein College of Medicine, Bronx, New York, USA, Bronx, New York

## Abstract

**Background:**

Previous literature has demonstrated an inverse relationship between the prevalence of parasitic infections and chronic inflammatory diseases such as allergies, inflammatory bowel diseases, and autoimmunity. The “Old Friends” and “Hygiene” Hypotheses posit that in the sanitized society of high-income countries, the lack of allergens and microbiota is causing immune dysregulation. This research has led some individuals to pursue unregulated "helminth therapies" - ingesting parasites to relieve autoimmune symptoms - despite the lack of FDA approval or controlled clinical trials for the marketed products. The growth of an unregulated market, with transactions often conducted via untraceable Bitcoin and without transparency of origin, poses a public health risk that requires awareness among infectious disease specialists.

**Methods:**

The researchers investigated 9 different helminth distributors to determine helminth species offered, origin, price, and “recommended dosage" of helminth therapy through the website Helminth Therapy Wiki. Helminth distributors analyzed include Symmbio, Au NAturel, Nathural, Tanawisa, Helminth Therapy NZ, Biome Restoration Ltd, The Llamas Clinic, YourSymbionts, and Autoimmune Therapies (AIT).

**Results:**

78% of the helminth providers sold *Necator americanus*, i.e. hookworms (NA), 22% sold *Trichuris trichiura*, i.e. whipworms (TTO), 22% sold *Hymenolepis diminuta*, i.e. rat tapeworms (HDC), and 11% sold *Trichuris suis*, i.e. pig whipworms (TSO). The price of one dose of these various parasites ranged from $120 USD to $1,789 USD, depending on species and provider. “Doses” were highly variable; the dose of NA ranged from 1 - 25 larvae, while TSO ranged from 500 - 2,500 larvae. Many companies used untraceable payment methods and contact preferences to maintain secrecy. Statements on several provider websites used inaccurate marketing, over-promised benefits, and under-emphasized risks.

**Conclusion:**

The unregulated market for helminth therapies poses significant public health and consumer protection issues, given the variability in doses, high costs, and dubious marketing claims. The lack of transparency and medical provider oversight further exacerbates the safety concerns in this market.

**Disclosures:**

**All Authors**: No reported disclosures

